# Correlation between serum factor VIII:C levels and deep vein thrombosis following gynecological surgery

**DOI:** 10.1080/21655979.2021.1981755

**Published:** 2021-11-30

**Authors:** Jiayi Liu, Cuiqin Sang, Zhenyu Zhang, Ying Jiang, Shuzhen Wang, Yao Li, Xiao Shi

**Affiliations:** a Department of Obstetrics and Gynecology, Beijing Chao-Yang Hospital, Capital Medical University, Beijing, China; b Department of Obstetrics and Gynecology, Beijing Daxing Hospital, Capital Medical University, Beijing, China

**Keywords:** Deep vein thrombosis, coagulation factor viii, coagulation factor viii:c

## Abstract

Deep vein thrombosis (DVT) is common in patients following gynecological surgery. Coagulation factor VIII (FVIII) is an important part of the human coagulation system, and FVIII:C is a component of FVIII with anticoagulant activity. 800 patients who underwent gynecological surgery were enrolled. General clinical data were harvested, and pre – and postoperative serum FVIII levels were determined. Lower-extremity ultrasound examination and/or postoperative pulmonary angiography were performed. Related data were analyzed statistically. DVT was the first manifestation of venous thromboembolism in all cases. There were a total of 46 cases, and the incidence of DVT was 5.8%. Progression to pulmonary embolism was confirmed in 16 cases, with an incidence of 2.0%. The independent risk factors for DVT after gynecological surgery were postoperative FVIII:C levels (odds ratio [OR] = 1.01), age (OR = 6.57), and operation time ≥3 hours (OR = 2.90) (*P* < 0.05). When the FVIII:C level was greater than the 75th centile (≥150 IU/dL), the risk of DVT was 2.99 times higher than that below the 25th centile (<100 IU/dL) (*P* < 0.05). When combined with the risk factor of operation time ≥3 hours, the risk increased to 3.17 times (*P* = 0.10). When combined with age ≥60 years, the risk was significantly increased, reaching 12.0 times (*P* < 0.05). Serum FVIII:C levels are an independent risk factor for DVT after gynecological surgery. Higher levels increase the risk of DVT after gynecological surgery, and they may have a dose-dependent relationship. A synergistic effect exists in combination with other risk factors, which further increases the risk.

## Introduction

Venous thromboembolism (VTE), which includes deep vein thrombosis (DVT) and pulmonary embolism (PE), is a venous reflux disorder resulting from thrombosis in the vein. Increased risk factors for thrombotic diseases result in a gradual increase in the proportion of gynecological postoperative VTE events. Based on a previous study, the incidence of DVT in patients without preventive measures ranges from 9.2% to 15.6%, and the incidence of PE in patients with DVT is 46.0%, which is higher than the incidence reported abroad [1]. Typical clinical manifestations of DVT of the lower extremities are swelling and pain in the unilateral lower extremity. However, there can be no obvious symptoms in the early stage, which is one of the reasons why it is easily overlooked. DVT can reduce quality of life; PE is associated with a high rate of disability and a mortality rate of up to 40% [2,3], which is a serious threat to the lives of patients. In addition, over the past decade, the incidences of DVT and PE have increased significantly.

The normal process of thrombosis depends on the combined action of the coagulation, anticoagulation, and fibrinolytic systems. The overall incidence of VTE has been reported to be 0.96%, the incidence of DVT is 0.71%, and the incidence of PE is 0.33%. Patients older than 60 years were also found to have a higher incidence of DVT in clinical practice. Abnormalities in certain steps can interrupt the balance of the entire system, resulting in pathological thrombosis. Coagulation factor VIII (FVIII) is a macromolecular glycoprotein whose low-molecular-weight component FVIII:C has coagulation activity. Previous researchers observed in animal studies that elevated serum FVIII:C levels can increase thrombosis, and some in vivo data have shown that FVIII may cause thrombosis by increasing the production and activity of thrombin and reducing protein C activity, the mechanism of which is not yet clear [4,5].

International large-scale prospective studies have shown that FVIII:C has a very close relationship with DVT, and an increased level of FVIII:C has been found to be an independent risk factor of DVT [6]. The risk of DVT is dose dependent [7]. The levels of FVIII:C may vary substantially depending on factors such as age, ethnicity, and genetics [8–10], and relevant statistical analysis has rarely been conducted in China. Therefore, we hypothesized that serum FVIII:C levels may be an independent risk factor for DVT after gynecological surgery. This study aims to investigate the relationship between serum FVIII:C levels and DVT after gynecological surgery and to explore the associated risks for predicting and evaluating DVT.

## Participants and methods

### 1. Participants

#### 1.1 Sampling source

All subjects enrolled in this study were patients who visited the gynecology department from May 2011 to December 2017 and required surgical intervention. All subjects were Chinese nationals, aged 18 to 86 years old. Patients provided signed informed consent before invasive examinations were conducted. All subjects cooperated well during the experiment.

#### 1.2 Inclusion criteria

1. Ultrasound (US) of both lower limbs confirmed there was no DVT before surgery.
2. Medical history, physical examination, and a panel of seven coagulation tests confirmed that there was no bleeding tendency before the operation.
3. No thromboembolic disease or hemorrhagic disorder had occurred within the preceding 6 months.
4. For patients using long-term anticoagulant medications, warfarin was stopped for at least 3 days before the operation, and low-molecular-weight heparin was stopped for at least 12 hours.
5. There were no absolute contraindications to surgery, and relative contraindications were well controlled.
6. The subject or his or her authorized representative provided an accurate medical history.
7. Informed consent form was signed.

#### 1.3 Exclusion criteria

1. VTE was detected before surgery.
2. Hemorrhagic tendency was diagnosed previously or identified for the first time, with a history of familial clustering of hemorrhagic disease.
3. Other conditions were present that may affect blood coagulation function within the past 6 months.
4. Severe cardiopulmonary dysfunction, shock, abnormal liver and kidney function, abnormal thyroid function, addictive drug abuse, mental abnormality, and so forth were present.
5. Anticoagulant drugs needed to be started as soon as possible after the operation because of comorbidities.
6. The subject refused to participate.

### 2. Methods

Clinical data were collected by the gynecologist, and history taking and physical examination were performed before the operation. Following the operation, the research staff obtained information on the operation method and operation time, continuously observed the subject, and inquired about lower-limb sensation and activity every day, or as necessary, as well as whether there were abnormal symptoms such as chest tightness, hemoptysis, or syncope.

Body fluid samples (i.e., fasting venous blood samples [for the blood coagulation test] as well as routine blood tests) were collected by experienced nurses before and after the operation. FVIII had to be collected within 12 hours after the operation. Serum FVIII:C levels were detected using the one-stage method. All operations complied with laboratory regulations and instrument reagent instructions.

A sonographer examined imaging data within 3 days before and after surgery. For patients in whom DVT was suspected outside this time period, the examination was repeated. If PE was suspected in the subject, pulmonary angiography was performed after an informed consent form was signed. The examination results were jointly issued by at least two imaging physicians.

### 3. Statistical analysis

SPSS software (22.0, IBM Company, Armonk, NY, USA) was used for data processing and statistical analysis. *P* < 0.01 was considered highly statistically significant, and *P* < 0.05 was considered statistically significant.

## Results

FVIII:C has been shown to have a very close relationship with DVT, and an increased level of FVIII:C is one of the independent risk factors of DVT. We suppose that serum FVIII:C levels may be an independent risk factor for DVT after gynecological surgery, and we therefore aimed to investigate the relationship between serum FVIII:C levels and DVT after gynecological surgery and to explore the associated risks for predicting and evaluating DVT.

### 4. Comparison of general clinical data

A total of 46 patients developed VTE after surgery, with an incidence of 5.8%. All cases presented first as DVT. Eighteen patients had symptoms of suspected PE, and two patients did not undergo computed tomography pulmonary angiography because they could not tolerate invasive examination. Only empirical anticoagulation treatment was administered, and the final incidence was 2.0%. The mean and chi-square results showed that the two groups differed in average age, operation time, preoperative and postoperative FVIII:C levels, whether they suffered from malignant tumors, and whether they suffered from hypertension (*P* < 0.01). Through further stratification by age, we found a significant difference between the population <60 years old and the population ≥60 years old (*P* < 0.01). There was no significant difference between US− and US+ in terms of body mass index (BMI), smoking history, or diabetes. Further comparison between BMI groups indicated no significant differences. See Table 1 for details.

1. Correlation of serum FVIII:C levels and DVT

**Table 1.** Comparison of general clinical data of Non-DVT Group Us – AND DVT Group Us+

	Us-		Us+			
	Cases	Mean±SD Or %	Cases	Mean±SD Or %	P Value(Dunnett)	
Before Operation FVIII:C(IU/dl)	727	94.22 ± 33.84	43	108.91 ± 39.99	<0.01	
Post Operation FVIII:C(IU/dl)	716	124.41 ± 39.01	44	143.52 ± 42.74	<0.01	
Age (years)	754	53.40 ± 9.80	46	63.15 ± 10.73	<0.01	
(1)<50	335	44.4	7	15.2	(1)vs.(2)0.16	
(2)50–60	264	35.1	12	26.1	(1)vs.(3)<0.01	
(3)60–70	102	13.5	13	28.3	(1)vs.(4)<0.01	
(4)≥70	53	7.0	14	30.4	(2)vs.(3)<0.01	
					(2)vs.(4)<0.01	
					(3)vs.(4)0.16	
BMI (Kg/m^2^)	754	25.05 ± 3.70	46	25.36 ± 3.91	0.58	
(1)<18.5	17	2.3	0	0.0	(1)vs.(2)0.53	
(2)18.5–24	377	50.0	21	45.7	(1)vs.(3)0.66	
(3)≥24	360	47.7	25	54.3	(2)vs.(3)0.77	
Operation time(Minute)	754	182.2 ± 89.3	46	256.9 ± 100.6	<0.01	
Smoking History	754		46			
Yes	12	1.6	2	4.3	0.17	
No	742	98.4	44	95.7		
Malignant Tumor	754		46			
Yes	236	31.3	24	52.2	<0.01	
No	518	68.7	22	47.8		
Hypertension	754		46			
Yes	219	29.0	23	50.0	<0.01	
No	535	71.0	23	50.0		
Diabetes	754		46			
Yes	108	14.3	10	21.7	0.17	
No	646	85.7	36	78.3		

Note: P < 0.01 was considered statistically significant.

#### 4.1 Correlation factors and risk factors of DVT

Correlation analysis indicated a weak correlation between age, operation time, and suffering from malignant tumors or hypertension (*P* > 0.01). There was no relationship between smoking history or diabetes (*P* > 0.05). See Table 2(1). Independent risk factors for DVT were postoperative FVIII:C levels (odds ratio [OR] = 1.01, 95% confidence interval [CI] = 1.00–1.02), age ≥60 years (OR = 6.57, 95% CI = 3.24–13.32), and operation time (OR = 2.90, 95% CI = 1.35–6.23). All results were statistically significant (*P* < 0.05). The OR value of age was the highest. See Table 2(2).

**Table 2.** (1) DVT risk factors analysis(Spearman Rank Correlation)

	ρ		P Value(two-tailed)	
Age≥60(years)	0.22		<0.01	
Operation time≥3(hours)	0.12		<0.01	
Smoking History Yes	0.05		0.17	
Malignant Tumor Yes	0.10		<0.01	
Hypertension Yes	0.11		<0.01	
Diabetes Yes	0.05		0.17	
Note: P < 0.01 was considered statistically significant.				
(2) DVT risk factors analysis				
		OR (95%CI)		P Value
Post Operation FVIII:C(IU/dl)		1.01 (1.00–1.02)		<0.05
Age≥60(years)		6.57 (3.24–13.32)		<0.05
Operation time≥3(hours)		2.90 (1.35–6.23)		<0.05
Smoking History Yes		2.69 (0.50–14.37)		0.25
Malignant Tumor Yes		1.40 (0.68–2.87)		0.37
Hypertension Yes		1.38 (0.69–2.77)		0.36
Diabetes Yes		1.08 (0.48–2.45)		0.85

Note: P < 0.05 was considered statistically significant.

#### 4.2 Incidence of DVT in individuals with low, moderate, and high serum levels of FVIII:C

Using the reference range of FVIII:C formulated by the laboratory department, 70 IU/dL and 150 IU/dL were set as the cutoff values, and the population was divided into three groups: G1 (low-level group, serum FVIII:C < 70 IU/dL), G2 (moderate-level group, serum FVIII:C < 150 IU/dL), and G3 (high-level group, serum FVIII:C ≥ 150 IU/dL). The results showed that the proportion of US+ increased gradually with the rise of FVIII:C levels in the general population. There were significant differences in the proportion among the three groups and between the two groups (*P* < 0.05). After stratifying by age, the proportion of US+ among the two groups increased with the rise in FVIII:C level. There was no DVT in group G1 of individuals younger than 60 years, and there was a significant difference in the proportion of US+ between group G3 and group G1 and between group G2 and group G1 (G1 vs. G2, *P* < 0.05; G1 vs. G3, *P* < 0.05). The intragroup and intergroup comparison showed no significant difference in the proportion of US+. See Table 3.

**Table 3.** Comparison of incidence of DVT in individuals with low, moderate and high serum levels of FVIII:C

	Cases	Us-(%)	Us+(%)	P Value (Dunnett)
General Population	760			<0.05
G1[Cases(%)]	49	48 (98.0)	1 (2.0)	G1vs.G2 < 0.10
G2[Cases(%)]	517	492 (95.2)	25 (4.8)	G1vs.G3 < 0.05
G3[Cases(%)]	194	176 (90.7)	18 (9.2)	G2vs.G3 < 0.10
Age <60(years)	574			<0.10
G1[Cases(%)]	41	41 (100.0)	0 (0.0)	G1vs.G2 < 0.05
G2[Cases(%)]	384	376 (97.9)	8 (2.1)	G1vs.G3 < 0.05
G3[Cases(%)]	149	141 (94.6)	8 (5.4)	G2vs.G3 0.27
Age ≥60(years)	186			0.31
G1[Cases(%)]	8	7 (98.0)	1 (2.0)	G1vs.G2 0.38
G2[Cases(%)]	133	116 (95.2)	17 (4.8)	G1vs.G3 0.49
G3[Cases(%)]	45	35 (90.7)	10 (9.3)	G2vs.G3 0.26

Note: Note: P < 0.01 was considered statistically significant.

#### 4.3 Risk of DVT in individuals based on quartile serum FVIII:C levels

We divided the general population into four groups based on serum FVIII:C levels: Q1 (serum FVIII:C < 100 IU/dL), Q2 (100 IU/dL ≤ serum FVIII:C < 124 IU/dL), Q3(124 IU/dL ≤ serum FVIII:C < 150 IU/dL), and Q4 (serum FVIII:C ≥ 150 IU/dL). After controlling for age and malignancy, the risk of DVT in the general population increased as the serum FVIII:C levels rose. The risk in the Q4 group (OR = 2.99, 95% CI: 1.27–7.99) was three times higher than that of the Q1 group (*P* < 0.05). After stratifying by surgery time, we noted an increased risk of DVT in both groups in accordance with the increase in FVIII:C levels. The OR of the Q4 group was 2.2 times that of the Q1 group in individuals with an operation time <3 hours, but the difference was not significant (*P* = 0.33); the risk in the Q4 group in individuals with an operation time ≥3 hours (OR = 3.17, 95% CI: 0.79–12.75) was 3.2 times that of the Q1 group, with a significant difference (*P* = 0.10). See Table 4.

**Table 4.** Risk analysis of interquartile levels of serum FVIII: C and DVT

	Cases	Us-(%)	Us+(%)	OR(95%CI)	P Value
General Population	760				
Q1[Caces(%)]	206	200 (97.1)	6 (2.9)	1.00	
Q2[Cases(%)]	180	174 (96.7)	6 (3.3)	1.20 (0.37–3.94)	0.76
Q3[Cases(%)]	180	166 (92.2)	14 (7.8)	2.24 (0.81–6.19)	0.12
Q4[Cases(%)]	194	176 (90.7)	18 (9.3)	2.99 (1.12–7.99)	<0.05
Operation time<3(hour)	420				
Q1[Cases(%)]	132	129 (97.7)	3 (2.3)	1.00	
Q2[Cases(%)]	117	115 (98.3)	2 (1.7)	0.78 (0.12–4.98)	0.79
Q3[Cases(%)]	93	89 (95.7)	4 (4.3)	1.76 (0.37–8.40)	0.48
Q4[Cases(%)]	78	73 (93.6)	5 (6.4)	2.15 (0.46–9.95)	0.33
Operation time≥3(hour)	340				
Q1[Cases(%)]	74	71 (95.9)	3 (4.1)	1.00	
Q2[Cases(%)]	63	59 (93.7)	4 (6.3)	1.93 (0.39–9.64)	0.42
Q3[Cases(%)]	87	77 (88.5)	10 (11.5)	2.23 (0.54–9.26)	0.27
Q4[Cases(%)]	116	103 (88.8)	13 (11.2)	3.17 (0.79–12.75)	0.10

Note: P < 0.05 was considered statistically significant.

#### 4.4 Incidence of DVT in individuals with extremely high serum FVIII:C levels

We set 200 IU/dL was as the cutoff value of FVIII:C, and the general population was divided into Gaserum FVIII:C < 200 IU/dL and Gb groups (serum FVIII:C ≥ 200 IU/dL), among which the Gb group had extremely high serum FVIII:C levels. After controlling for malignant tumors, the incidence of DVT in the Gb group was significantly higher than that of the Ga group, and the risk of DVT (OR = 3.01, 95% CI: 1.08–8.36) was three times that of the Ga group, with a significant difference (*P* < 0.01). After age stratification, there was no significant difference in the incidence or risk of DVT between the Ga and Gb groups in the population younger than 60 years. The incidence of DVT in the Gb group was higher than that of the Ga group, and the risk (OR = 12.00, 95% CI: 2.00–72.03) was 12.0 times that of Ga group, with a significant difference (*P* < 0.05). See Table 5.

**Table 5.** Incidence of DVT in individuals with extremely high serum FVIII:C

	Cases	Us-(%)	Us+(%)	OR(95%CI)	P Value
General Population	760				
Ga[Cases(%)]	729	690 (94.7)	39 (5.3)	1.00	
Gb[Cases(%)]	31	26 (83.9)	5 (16.1)	3.01 (1.08–8.36)	<0.01
Age <60(years)	574				
Ga[Cases(%)]	549	534 (97.3)	15 (2.7)	1.00	
Gb[Cases(%)]	25	24 (96.0)	1 (4.0)	1.30 (0.16–10.39)	0.80
Age ≥60(years)	186				
Ga[Cases(%)]	180	156 (86.7)	24 (13.3)	1.00	
Gb[Cases(%)]	6	2 (33.3)	4 (66.7)	12.00 (2.00–72.03)	<0.05

Note: P < 0.05 was considered statistically significant.

### 5. Risk factors of increased serum FVIII:C levels

The cutoff value of increased serum FVIII:C was set as 150 IU/dL, and we analyzed the physiological factors that might lead to the rise. We compared the clinical data of the two groups, and we found a significant difference in the proportion of malignant tumors (*P* < 0.01) but no significant difference in overweight, smoking history, diabetes mellitus, and age. The results of logistic regression analysis showed that malignancy was an independent risk factor of increased serum FVIII:C (OR = 1.66, 95% CI: 1.14–2.39; *P* < 0.01), but overweight, aging, smoking history, and diabetes mellitus were not (*P* > 0.05). See Table 6.

**Table 6.** Risk factors of increased serum FVIII:C

	FVIII:C < 150IU/dl Cases(%)	FVIII:C ≥ 150IU/dl Cases(%)	P Value1	OR (95%CI)	P Value2
Malignant Tumor	566	194	<0.01	1.66 (1.14–2.39)	<0.01
Yes	155 (27.4)	86 (44.3)			
No	411 (72.6)	108 (55.7)			
BMI≥24 (Kg/m^2^)	566	194	0.79	1.04 (0.73–1.47)	0.84
Yes	338 (59.7)	118 (60.8)			
No	228 (40.3)	76 (39.2)			
Smoking History	566	194	0.33	0.51 (0.11–2.34)	0.39
Yes	12 (2.1)	2 (1.0)			
No	554 (97.9)	192 (99.0)			
Diabetes	566	194	0.34	0.76 (0.46–1.25)	0.27
Yes	89 (15.7)	25 (12.9)			
No	477 (84.3)	169 (87.1)			
Age ≥60 (years)	566	194	0.63	1.03 (0.69–1.55)	0.87
Yes	141 (24.9)	45 (23.2)			
No	425 (75.1)	149 (76.8)			

Note: P < 0.05 was considered statistically significant;P Value1: ANOVA; P Value2: Logistic Regression.

## Discussion

### 1. FVIII features and testing methods

FVIII is a macromolecular glycoprotein with a molecular weight of about 1 to 2 million. Its structure includes two parts: a low-molecular-weight component (FVIII:C with coagulation activity) and a high-molecular-weight component (containing FVIII antigen FVIIIR: Ag, antigen; VWF, VW factor VIIIR). FVIII is an important part of the coagulation cascade. Together with calcium ions and phospholipids, FVIII acts as a cofactor for activated factor IX and forms an endogenous X enzyme with it, reacts with factor X, and activates downstream prothrombin to produce thrombin. Endogenous X enzyme is located on the common pathway of the intrinsic and extrinsic coagulation pathways and can produce a ‘waterfall effect’ due to reverse activation of itself and downstream products. There is great variation in individual plasma FVIII levels, and more than half of the variation is genetically determined [11]. Plasma VWF combines with FVIII to form VWF–FVIII, which stabilizes the FVIII heterodimer, avoiding its premature hydrolysis by protease and prolonging its plasma half-life (8–12 hours). On the other hand, the half-life of free FVIII (unbound FVIII) is significantly shorter [12], which demonstrates how the half-life of FVIII depends mainly on the clearance rate of VWF. Other influencing factors include ABO blood type (the levels in normal O-type individuals are 25%–30%, which are lower than those in non–O-type individuals) [13], age (increases with age), female (higher than male), exercise, stress, pregnancy status, surgery, and other stress responses (such as chronic inflammation or malignant tumors) [14,15].

### 2. Incidence and risk factors of VTE

John et al [16] conducted a large clinical retrospective study that included 1,432,855 patients who underwent surgery under general anesthesia. The overall incidence of VTE was 0.96%, the incidence of DVT was 0.71%, and the incidence of PE was 0.33%. In multivariate logistic regression analysis, gynecological surgery was a negative factor for overall risk (OR = 0.68, 95% CI: −0.52 to −2.3; *P* < 0.001). Joseph et al [17] studied DVT in 1974 Caucasian patients after gynecological surgery and found an overall incidence of 1.9%, of which the incidence was 4.2% in the group with malignancy and 0.2% in the group with benign disease. VTE has long been considered uncommon among Asians. Yuk Law et al [18] conducted an epidemiological survey in an ethnic Han Chinese population of 7.1 million that showed that, the incidence of DVT after all operations was 0.2% and the incidence of PE was 0.12%; the incidence of DVT after obstetric and gynecological surgery was 0.13%, and the incidence of PE was 0.03%, which was a significant increase as compared with findings 10 years previously. Qu et al [19] found that the incidence of DVT in patients without thrombosis prevention measures after gynecological surgery in China was 9.2% to 15.6%, and the incidence of PE in those with DVT was 46%, which was much higher than the estimates in studies abroad.

Many studies have been conducted on VTE risk factors. The factors of long operation time and advanced age found in this study have been repeatedly verified as independent risk factors for DVT and are included in various VTE risk-scoring systems. This study did not find a link between BMI and DVT, but Abdollahi et al [20] studied the relationship between body weight and coagulation factor levels in patients with DVT and found that overweight was a risk factor for DVT. Obesity (BMI ≥ 30 kg/m^2^) doubles the risk of disease. In obese people, serum FVIII and FIX levels were also increased, but after controlling for influencing factors, obesity itself remained a risk factor. The study also found that in women with a BMI >25 kg/m^2^, the use of oral contraceptives and obesity had a synergistic effect, further increasing the risk of thrombosis. Fifty percent of US+ patients had hypertension, which is a common comorbidity. John et al [16] conducted a univariate analysis of many influencing factors and found that the effect of hypertension on the risk of DVT was not prominent (OR = 0.93, 95% CI: −0.11 to −0.03; *P* < 0.001). However, in certain populations, hypertension showed a strong influence on DVT. Li Qun et al [21] explored the risk factors of DVT in patients with gynecological malignancies and found that hypertension was one of the independent influencing factors (OR = 2.638, 95% CI: 1.523–4.539, *P* < 0.0001). A meta-analysis conducted by Ageno et al [22] found that the VTE risk was increased in patients with classic cardiovascular disease risk factors, including obesity (OR = 2.3), high blood pressure (OR = 1.5), diabetes (OR = 1.4), and hyperlipidemia (OR = 1.2). The findings from various studies suggest that the impact of comorbidities on VTE is complex, and it is necessary to explore their intrinsic factors and elucidate clearer risk prediction indicators.

### 3. Correlation between different levels of FVIII and VTE and their associated risks

In 1995, the Leiden Thrombophilia Study first reported the correlation between high levels of serum FVIII and VTE and found that FVIII:C was the only independent risk factor in the study. VWF and non–O blood groups were associated with an increased risk of VTE by their effects on FVIII:C. A number of subsequent follow-up cohort studies and case-control studies have confirmed that the increased incidence of DVT and PE is associated with increased FVIII:C levels.

There is a dose-dependent relationship between FVIII levels and VTE risk. Ota et al [6] retrospectively analyzed the relationship between serum FVIII levels and the risk of VTE among patients with VTE. The study included 68 subjects and 40 healthy controls matched by age and gender, and blood samples were collected at least 3 months after the acute phase of VTE, for which anticoagulant therapy had been given. The ‘one-stage method’ was used to measure FVIII activity. We found that when the cutoff value was the 25th centile, the conferred OR was 2.5 when the cutoff value was the 50th centile, the conferred OR was 5.2 times; when the cutoff value was at the 75th centile, the OR increased dramatically to 32. Kraaijenhagen et al [23] studied the relationship between serum FVIII levels and the risk of single-episode DVT and recurrent DVT and found that for every 10-IU/dL increase in FVIII:C, the risk of VTE in single-episode and recurrent DVT increased by 10% and 24%, respectively. These results demonstrate that the degree of risk changed continuously with the change in FVIII levels; that is, there was a dose-dependent relationship.

The reason for dose dependence might be related to FVIIIa increasing IXa activity, which in turn increases Xa, promoting thrombin and fibrin production [24]. Ryland et al [25] studied the relationship between FVIII, D dimer, and thrombin generation test (TGT) parameter changes in patients with a history of VTE after at least 4 weeks of anticoagulation treatment. The study included 91 subjects and 52 healthy controls. These authors determined FVIII:C levels using the one-stage method and the colorimetric method and, obtained the relevant TGT parameters. The results showed that the normalized endogenous thrombin potential (nETP) and peak value (nPeak) of the subjects were greater than those of controls, and the differences were highly significant (*P* < 0.001); that is, the subjects showed stronger clotting tendency. When FVIII was greater than 200 IU/dL, the average nPeak and average nETP were higher than when FVIII was less than 150 IU/dL (*P* < 0.05). Subsequently, the dose-response relationship between TGT and FVIII levels was explored. Plasma samples with FVIII levels of 150 IU/dL, 200 IU/dL, 300 IU/dL, and 400 IU/dL were established, and standard plasma was used as a control to perform in vitro TGT. The results revealed that the higher the FVIII level, the shorter the thrombin generation lag time, and the higher the peak, the larger the ETP (the plateau effect occurs when FVIII reaches 200–300 IU/dL). The hypercoagulable state of patients with VTE was related to the level of FVIII, and higher levels were associated with an increase in the generation rate and amount of thrombin, making the relationship dose dependent. In any case, because of the limitation of the total amount of prothrombin, the increase in thrombin generation would promote thrombosis.

The conclusions drawn in this study suggest that as the levels of FVIII increase, the incidence of DVT increases, but the identification of a clear dose-dependent relationship was lacking. Because it is easier to show the impact on risk with higher levels of FVIII, the ideal method of stratification in this experiment would be to establish three thresholds of 90th, 95th, and 99th, to focus on exploring the incidence in a population with high levels of FVIII. However, because the sample size was limited, only the situation in which the serum FVIII level was ≥200 IU/dL (95th) can be discussed. We found that the incidence of DVT greater than this level was three times higher than that lower than this level. After matching for age, the risk was increased by 12 times in elderly patients, but such an increase was not observed among nonelderly patients. This finding is worthy of attention for clinical workers.

### 4. Other issues

8In addition to expanding the sample size and conducting multicenter studies, recent studies on the relationship between serum FVIII levels and VTE have extended the follow-up. Long-term follow-up has allowed for assessing changes in FVIII levels over an extended period of time. FVIII and VTE have both a vertical dose-dependent relationship and a horizontal temporal relationship. The relationship between long-term FVIII levels and the incidence of VTE and related complications remains to be explored. All patients who undergo gynecological surgery are female. The characteristics of the female population should be considered when discussing the incidence and risk factors, and real-world factors need to be taken into account when conducting the design and analysis of the experiment. The use of oral contraceptives for women of childbearing age is considered one of the risk factors for VTE. Postmenopausal women rarely use such drugs, but the medicines containing estrogen for perimenopausal syndrome replacement therapy may also increase the risk of VTE. The usage rate of these drugs in different regions also varies and is typically more common in the West and developed regions. It has been reported that smoking is a risk factor for VTE, but no conclusions in this respect were drawn in this study. Perhaps this is because of the low rates of smoking in the female population of this area or the concealment of personal history, but increasing the sample size and conducting a multicenter investigation would be necessary. It is more meaningful to discuss lifestyle features (such as diet and exercise habits) and blood lipid levels than to directly analyze BMI. Radiochemotherapy is a common treatment for gynecological tumors. It has been reported that radiotherapy is an independent risk factor for thrombosis in patients with tumor [21]. Recording radiotherapy-related information and analyzing the risk of thrombosis can help prevent thrombosis and improve the quality of life of patients receiving radiotherapy.

## Conclusion

Serum FVIII:C levels are an independent risk factor for DVT after gynecological surgery. Higher levels increase the risk of DVT after gynecological surgery, and they may have a dose-dependent relationship. A synergistic effect exists in combination with other risk factors, which further increases the risk.

## Highlights

1. Serum FVIII:C levels are an independent risk factor for DVT after gynecological surgery.

2. Higher levels increase the risk of DVT after gynecological surgery.

3. There is a dose-dependent relationship between serum FVIII:C levels and DVT.

4. A synergistic effect in combination with other risk factors further increases the risk.
